# Protocol for design, construction, and selection of genome phage (gPhage) display libraries

**DOI:** 10.1016/j.xpro.2021.100936

**Published:** 2021-11-08

**Authors:** Luis Antonio Rodriguez Carnero, André Azevedo Reis Teixeira, Fenny Hui Fen Tang, Andréia Kuramoto, Maria Júlia Manso Alves, Walter Colli, João Carlos Setubal, Edécio Cunha-Neto, Renata Pasqualini, Wadih Arap, Ricardo José Giordano

**Affiliations:** 1 Department of Biochemistry, Institute of Chemistry, University of São Paulo, São Paulo, SP 05508, Brazil; 2 Rutgers Cancer Institute of New Jersey, Newark, NJ 07101, USA; 3 Heart Institute (InCor), University of São Paulo School of Medicine, São Paulo, SP 05403, Brazil; 4 Division of Cancer Biology, Department of Radiation Oncology, Rutgers New Jersey Medical School, Newark, NJ 07103, USA; 5 Division of Hematology/Oncology, Department of Medicine, Rutgers New Jersey Medical School, Newark, NJ 07103, USA; 6 Division of Clinical Immunology and Allergy, University of São Paulo School of Medicine, São Paulo, SP 01246, Brazil; 7 Institute for Investigation in Immunology, University of São Paulo School of Medicine, São Paulo, SP 01246, Brazil

**Keywords:** Sequence analysis, Genomics, Sequencing, High Throughput Screening, Immunology, Microbiology, Model Organisms, Molecular Biology, Antibody, Molecular/Chemical Probes

## Abstract

This protocol describes the genomic phage (gPhage) display platform, a large-scale antigen and epitope mapping technique. We constructed a gPhage display peptide library of a eukaryotic organism, *Trypanosoma cruzi* (causative agent of Chagas disease), to map the antibody response landscape against the parasite. Here, we used an organism with a relatively large but intronless genome, although future applications could include other prevalent or (re)emerging infectious organisms carrying genomes with a limited number of introns.

For complete details on the use and execution of this protocol, please refer to Teixeira et al. (2021).

## Highlights

• A streamlined protocol to build genome shotgun phage display libraries
• Scalable to different organisms, eukaryotes or prokaryotes
• Optimizes key steps for high throughput identification of antibody antigens/epitopes
• gPhage is also a platform to study conformational and difficult-to-validate antigens

## Graphical abstract

**Figure:**
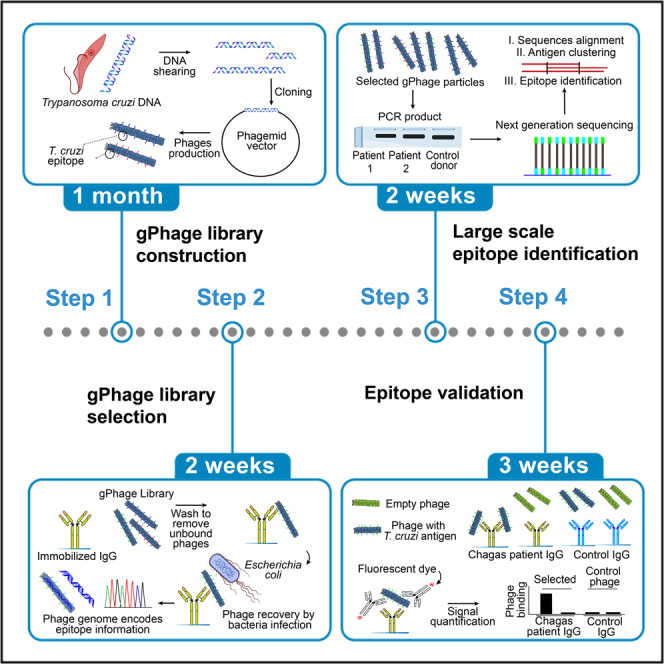

## Before you begin

### Phagemid vector selection and production

**Timing: 2 days**

For the gPhage display peptide library generation and production we recommend the use of the phagemid vector pG8SAET (Zhang et al., 1999) or a functional equivalent. This vector (available upon request) is particularly suitable for this work because it contains a unique site for a blunt-end restriction enzyme (*Eco105I*), which allows for cloning DNA inserts generated by random fragmentation methods. It will generate phage particles displaying hundreds of peptides (∼400–500 copies) fused to the recombinant major capsid protein VIII (rpVIII) as opposed to the single-digit display of peptides (∼1–5 copies) obtained when using the minor capsid protein III (pIII), thereby potentially increasing biochemical avidity by up to two orders of magnitude.

1. Transform *Escherichia coli* DH10B strain with pG8SAET plasmid.
2. Grow the cells in 500 mL LB broth (50 μg/mL carbenicillin) at 37^o^C for 16 h with agitation at 250 revolutions per minute (rpm).
3. On the next day, perform initial plasmid DNA extraction with a standard kit (see below).
4. Optional: We recommend an additional purification step with cesium chloride (CsCl) density gradient to improve vector purity, increase ligation efficiency and minimize contamination with *E. coli* genomic DNA (Sambrook and Russel, 2001).

***Note:*** We recommend Plasmid Maxi Kit (QIAGEN, 12162) according to manufacturer instructions for the initial plasmid purification prior to the additional ultracentrifugation-based plasmid purification with a CsCl density gradient.

### DNA extraction from *Trypanosoma cruzi*

**Timing: 8 days (7 days for epimastigote culture, 1 dayforDNA extraction)**

As the source of genomic diversity for the library, one must obtain genomic DNA from the organism of interest. The protocol should be optimized depending on the organism of interest. Here we illustrate the protocol used to construct a *T. cruzi* genomic library. It is worth mentioning that most *T. cruzi* genes do not carry introns and almost half of its genome is comprised of coding sequences (El-Sayed et al., 2005). One must take these into account when selecting a model organism because antigens or epitopes that span two or more exons will likely not be correctly displayed in the library design reported here.

5. Grow *T. cruzi* epimastigotes at 28°C in LIT (liver infusion-tryptose) medium supplemented with heat-inactivated 10% cell culture grade Fetal Bovine Serum (FBS) (Alves et al., 1986; Giordano et al., 1999).
6. Centrifuge 200 mL of the saturated culture containing approximately 10^9^ cells/mL at 1,000 × *g* (centrifugal force) at 23°C–25°C for 10 min and wash them three times with phosphate-buffered saline (PBS). It will take approximately 1 week (depending on the strain) for the epimastigote culture to reach the lag-phage.
7. Resuspend the cells in 20 mL lysis buffer containing 1% sodium dodecyl sulfate (SDS) and proteinase K (100 μg/mL).
8. Incubate at 50°C for 2 h. Centrifuge the suspension and recover the supernatant.
9. Add 1:1 phenol:chloroform (vol/vol). Homogenize it by using a vortexing apparatus and centrifuge the admixture for 5 min at 7,000 × *g* at 23°C–25°C.
10. Repeat the previous step until solids are no longer observed in the interface between the aqueous (lower) and organic (upper) phases.
11. Carefully recover the aqueous phase. Add 0.1 volumes of 3M sodium acetate pH 5.2 and 2 volumes of ice-cold ethanol. Incubate it at −20°C for 15 min.
12. Precipitate the DNA by centrifuging at 20,000 × *g* for 15 min at 4°C. Wash the pellet of DNA with 70% ethanol and gently re-suspend the genomic DNA in 2 mL TE buffer (10 mM Tris pH 8, 1 mM EDTA).

***Note:*** We used ∼2 mg of genomic *T. cruzi* DNA in order to obtain a gPhage library with ∼10^8^ transformants.

### IgG purification from selected donors

**Timing: 3 days**

13. Serum sample selection from donor patients and controls. A cohort of patients should be selected according to the aims of the study and the particulars of each disease (or infectious agent) of interest. As an example, we selected 80 donors including patients with Chagas disease (N=60) plus control healthy volunteer donors (N=20) with the following guidelines:
  a. Candidate Chagas disease patients with at least two positive results for the presence of anti-*T. cruzi* antibodies.
  b. All candidate patients underwent electrocardiography (EKG) and echocardiography (Echo) and those with abnormal EKG (either right bundle branch block or left anterior fascicular block) were classified as having either mild cardiomyopathy, when the Echo ejection fraction of the left ventricle (LVEF) was higher than 40% (LVEF> 40%), or severe cardiomyopathy, when ejection fraction of the left ventricle was lower or equal 40% (LVEF ≤ 40%).
  c. Patients with no EKG alterations were deemed asymptomatic.
  d. Serum samples were pooled into cohorts of 10 donors to form two independent sets (biological duplicates) for each disease condition: control (2 × 10 donors), asymptomatic (2 × 10 donors), mild cardiomyopathy (2 × 10 donors) and severe cardiomyopathy (2 × 10 donors).
14. IgG was then purified from the pool of serum samples of each cohort by using affinity chromatography with Protein-G Sepharose (GE Healthcare), according to the manufacturer instructions.
15. At this point it is important to check the quality of the purified IgG by SDS-PAGE and then quantify protein concentration for each pool using the Bradford assay (or an equivalent alternative method for protein quantification). The resulting purified IgG may be kept frozen (at −20°C or less) in small individual aliquots to be thawed out when the gPhage display selection is performed. IgG concentration should be at least 50–100 μg/mL with purity greater than 90%.

## Key resources table

**Table:** 

REAGENT or RESOURCE	SOURCE	IDENTIFIER
**Antibodies**		

Rabbit anti-bacteriophage IgG (1:400)	Sigma	Cat#B7786-5μL
Goat anti-rabbit IgG IRDye 680 LT (1:1000)	LI-COR Biosciences	Cat#926-68021
Goat Anti-Human IgG, Fc fragment specific	Jackson ImmunoResearch	Cat#109-001-008

**Bacterial and virus strains**		

*Escherichia coli* strain DH10B	Thermo Fisher Scientific	Cat#EC113
*Escherichia coli* strain TG1	Lucigen	Cat#60502-1
M13K07 Helper Phage	New England BioLabs	Cat#N0315 S

**Biological samples**		

Donor serum samples	Heart Institute, University of Sao Paulo. Approved by the Institutional Review Board at the University of São Paulo Medical School, Brazil. Approval number 0265/10. For more details of the subjects, refer to Teixeira et al. (2021).	N/A
Bovine blood	Certified commercial slaughterhouse or laboratory vendor.	N/A

**Chemicals, peptides, and recombinant proteins**		

Phosphate buffered saline	Thermo Fisher Scientific	Cat#21600010
Sodium dodecyl sulphate	Bio-Rad Laboratories	Cat#1610301
Proteinase K Solution	Thermo Fisher Scientific	Cat#AM2548
Phenol:Chloroform:Isoamyl Alcohol 25:24:1	Sigma	Cat#P3808
Ethanol Absolute	Merk	Cat#1009831000
UltraPure Tris Buffer	Thermo Fisher Scientific	Cat#15504-020
EDTA	Sigma	Cat#E5134
T4 DNA polymerase	Thermo Fisher Scientific	Cat#EP0062
Tryptone	Thermo Fisher Scientific	Cat#LP0042
Yeast extract	Thermo Fisher Scientific	Cat#LP0021
Dipotassium hydrogen phosphate	Merck	Cat#5101
Monopotassium phosphate	Sigma	Cat#V003710
Disodium phosphate heptahydrate	Synth	Cat#F1031.01.AH
LB Broth	Sigma	Cat#L3022-1Kg
LB agar	Sigma	Cat#L2897-1KG
Triptose broth	Difco	Cat#262200
Carbenicillin	Sigma	Cat#C1389-5G
Penicillin	Sigma	Cat#D7794-100MU
Streptomycin	Sigma	Cat#S9137-100G
CsCl	Sigma	Cat#V000561
Ethidium bromide	Thermo Fisher Scientific	Cat#15585-011
Eco105I	New England BioLabs	Cat#R0130L
FastAP	Thermo Fisher Scientific	Cat#EF0651
Low-melting point agarose	Sigma	Cat#A9539-500G
T4 DNA ligase	Thermo Fisher Scientific	Cat#EL0011
Polyethylene Glycol 8000	Amresco	Cat#0159-1Kg
Sodium chloride	Merck	Cat#1064041000
Potassium chloride	Synth	Cat#C1060.01.AH
Glycerol	Sigma	Cat#G5516
Glucose	Sigma	Cat#S9137-100G
Protein G Sepharose 4 Fast Flow	GE Healthcare	Cat#17-0618-02
Bovine Serum Albumin	Sigma	Cat#A2153-1kg
Liver Infusion Broth	Difco	Cat#226920
Fetal Calf Serum	Vitrocell	Cat#SOROFETAL500
Carbonate	Merck	Cat#106392
Tween-20	Sigma	Cat#V001280
Synthetic epitope peptides	Chinese Peptide Company (China)	N/A

**Critical commercial assays**		

QIAGEN Plasmid Maxi Kit	Qiagen	Cat#12162
QIAprep Spin Miniprep Kit	Qiagen	Cat#27104
Taq DNA Polymerase (5 U/μL)	Thermo Fisher Scientific	Cat#EP0401
KAPA HiFi HotStart ReadyMix Kit	Roche	Cat#7958935001
QIAquick PCR Purification Kit	Illumina	Cat#FC-131-1024
Nextera XT DNA Library Preparation Kit (24 samples)	Qiagen	Cat#28106
KAPA Library Quantification Kit	Roche	Cat#KR0405
MiSeq Reagent Kit v2 (500-cycles)	Illumina	Cat#MS-102-2003
SIGMAFAST OPD	Sigma	Cat#P9187

**Experimental models: Organisms/strains**		

*Trypanosoma cruzi* epimastigotes Sylvio-X10 c11 (Pará, Brazil)	Dr. Bianca Zingales, Chemistry Institute, University of São Paulo, Brazil (Zingales et al., 2009)	N/A

**Oligonucleotides**		

Illumina sequencing oligonucleotides	Exxtend (Brazil)	N/A

**Recombinant DNA**		

pG8SAET	Department of Microbiology Swedish University of Agricultural Sciences Uppsala University, Sweden (Zhang et al., 1999)	GenBank: AF130864.1https://www.ncbi.nlm.nih.gov/nuccore/4754815
gPhage library	This article	N/A

**Software and algorithms**		

BLAST	https://ftp.ncbi.nlm.nih.gov/blast/executables/blast+/	v.2.12.0
PEAR	https://cme.h-its.org/exelixis/web/software/pear/doc.html	v 0.9.10
MAFFT	https://mafft.cbrc.jp/alignment/software/	v 7.307
Jalview	https://www.jalview.org/	v 2.11.0
MUSCLE	https://www.ebi.ac.uk/Tools/msa/muscle/	3.8.31
Chimera	https://www.cgl.ucsf.edu/chimera/	v 1.11
XSTREAM	https://amnewmanlab.stanford.edu/xstream/	v 1.73
FuzzyWuzzy	https://github.com/seatgeek/fuzzywuzzy	v 0.18
HMMER	http://hmmer.org/download.html	3.1b2
Sequence analysis script	This article	Data S1
Clustering script	This article	Data S2

**Other**		

96 Well EIA/RIA High Binding Plate	Corning Incorporated Costar	Cat#3590

## Materials and equipment

**Table:** LIT medium

Reagents	Final concentration	Amount
Triptose broth	5 g/L	5g
Sodium chloride	4 g/L	4g
Potassium chloride	0.4 g/L	0.4 g
Disodium phosphate heptahydrate	15 g/L	15 g
Penicillin G	0.15 g/L	0.15 g
Glucose	2 g/L	2 g
Streptomycin	0.15 g/L	0.15 g
Liver infusion broth	5 g/L	5 g
Bovine blood	-	20 mL
Cell culture grade, heat inactivated FBS	10%	100 mL

To prepare 1 L of medium. Dissolve all the reagents except the bovine blood in 800 mL of double-distilled water (ddH_2_O). Incubate at 37^o^C for 1 h. Cool the solution on ice. Centrifugate 20 mL of bovine blood at 12,000 × g for 10 min at 23°C–25°C and add the supernatant to the medium. Adjust pH to 7.3, complete volume to 900 mL with ddH_2_O and sterilize it by filtration (0.22 μm). Aliquot and store medium at 4^o^C. Before use, add 100 mL of heat-inactivated FBS under sterile conditions.

**Table:** Lysis Buffer

Reagents	Final concentration	Amount
Tris-HCl pH 8.0 stock solution	100 mM	50 mL
Sodium dodecyl sulfate 10% stock solution	1%	50 mL
Proteinase K Solution (20 mg/mL)	100 μg/mL	2.5 mL

For 500 mL, mix 300 mL of ddH_2_O with 50 mL of Tris-HCl 1M pH 8.0 stock solution and 50 mL of 10% SDS stock solution. Adjust pH to 8.0 with NaOH or HCl 1M and complete with ddH_2_O to the final volume. Store at RT. Before use, add proteinase K to a final concentration of 100 μg/mL.

TE Buffer

For TE buffer, use 10 mM Tris-HCl pH 8.0 containing 1 mM EDTA.

For 500 mL, mix 400 mL of ddH_2_O with 5 mL of Tris 1 M pH 8.0 stock solution and 1 mL of 0.5 M EDTA stock solution. Check pH and if necessary, adjust pH to 8.0 with NaOH or HCl 1M and complete to 500 mL with ddH_2_O. Store at 23°C–25°C until use.

**Table:** TB: phosphate broth

Reagents	Final concentration	Amount
Yeast extract	24 g/L	24 g
Tryptone	20 g/L	20 g
Glycerol	4 mL/L	4 mL
Phosphate buffer (0.17 M KH_2_PO_4_, 0.72 M K_2_HPO_4_)	0.017 M KH_2_PO_4_ 0.072 M K_2_HPO_4_	100 mL

Dissolve yeast extract, tryptone and glycerol in 900 mL of ddH_2_O and sterilize the solution (autoclave). After cooling the admixture, add the phosphate buffer under sterile technique. Store the solution at 23°C–25°C for 6 months.

**Table:** PEG/NaCl

Reagents	Final concentration	Amount
PEG 8000	166,7 g/L	100 g
NaCl	3,3 M	116.9 g

To obtain a 600 mL solution, dissolve polyethylene glycol (PEG) 8,000 and NaCl with 450 mL ddH_2_O. Once dissolved, complete to 600 mL final volume with ddH_2_O. Sterilize (autoclave) and mix thoroughly while cooling to avoid phase separation. Store solution at 4°C until use. Discard if any precipitation is observed.

## Step-by-step method details

### gPhage library construction

**Timing: 1 month**

**Figure 1:**
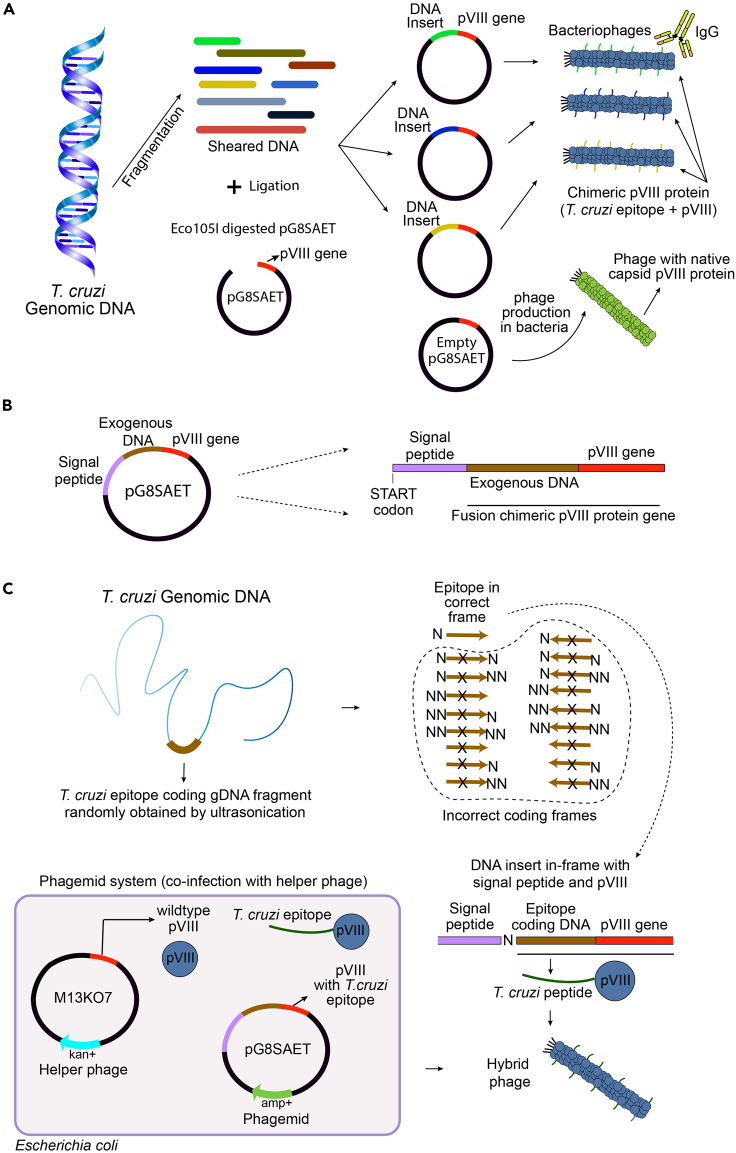
Constructing a gPhage library (A) To build the gPhage library, genomic DNA is fragmented and cloned into a phagemid vector such as pG8SAET. *Escherichia coli* is transformed with the vector and subsequently used to produce the gPhage library displaying the epitopes that can be recognized by the corresponding donor-derived and control-derived immunoglobulins. (B) Detail of the fusion transcript encoded by modified pG8SAET. The construct is formed by a signal peptide, the epitope-encoding DNA and the rpVIII gene. When transformed into bacteria, pG8SAET will produce a fusion rpVIII protein, as described. (C) After fragmentation and cloning, only 1 in 18 inserts will be in the correct frame (i.e., frame 2) to yield a phage particle displaying a *T. cruzi*-derived peptide. In the correct frame, the mature constructed protein will be formed by the epitope fused in-frame to the rpVIII. For using the phagemid system, co-infection with a helper phage allows the production of native pVIII protein and the other bacteriophage proteins while the pG8SAET phagemid encodes the rpVIII displaying the epitope. These two pVIII proteins (i.e., native and recombinant) will be packed into a hybrid phage construct.

**Figure 2:**
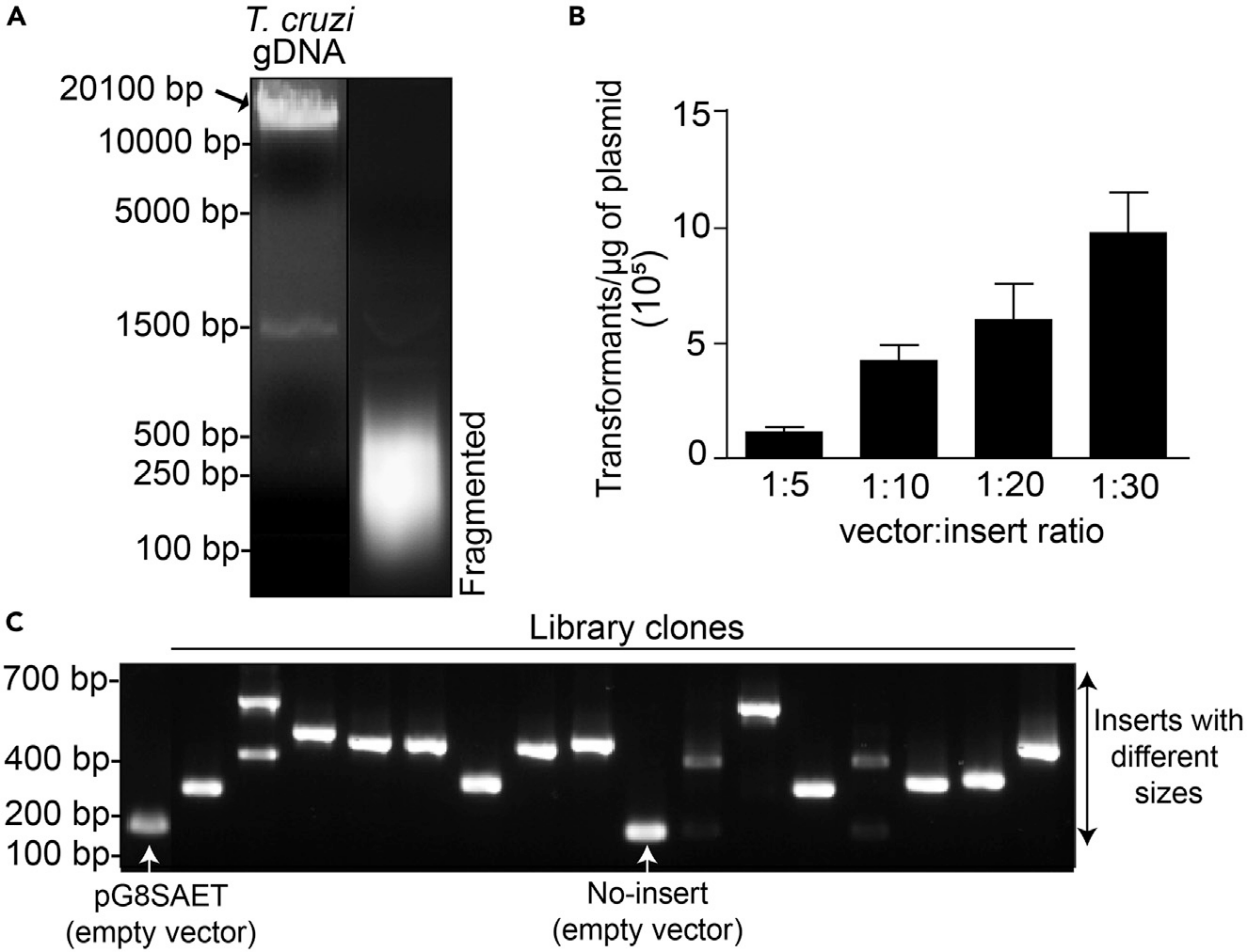
Building and quality control of a *Trypanosoma cruzi* genomic DNA library (A) *T. cruzi* genomic DNA can be fragmented with the COVARIS S2 ultrasonicator. The image shows an agarose gel electrophoresis comparing *T. cruzi* DNA before and after fragmentation. The obtained fragments were mainly distributed between 100-500 bp. (B) The efficiency of ligation depends on the vector:insert ratio. In the example with *T. cruzi* fragmented DNA and pG8SAET, the 1:30 ratio yielded the best results. Error bars represent the standard error of the mean (SEM, N=3) (C) After a PCR amplification of individual clones with primers flanking the insert, each clone will show a particular product size. When using a molecular ladder to calculate the molecular weight of the products, this value serves to estimate the coverage of the library. As a technical cautionary note (shown), a minor subset of clones will have an empty vector (non-insert), with the same size as pG8SAET.

The gPhage display library is constructed in three sequential steps: (i) insert preparation (fragmenting the genomic DNA to the desired size range and making it suitable for cloning), (ii) cloning the purified DNA inserts into the phagemid vector and transformation into bacteria, and (iii) producing phage particles from the library by using a helper phage (Figure 1). We recommend preparing inserts between 100-500 bp (Figure 2A) because this size range allows for the identification of linear and conformational epitopes that may still be fully sequenced [Illumina Miseq 2**×**250 bp; Teixeira et al. (2021)]. Please see Figure 1 in this article for other cloning logistics and details of the gPhage display library construction strategy for *T. cruzi*: DNA insert size, vector, possible orientation and reading frames of inserts, and genomic coverage distribution.

1. Blunt-ending of genomic DNA fragments
  Purified genomic DNA may be subjected to fragmentation in a COVARIS S2 equipment (Covaris Inc), which yields fragments in the desired size range in a reproducible manner. If access to such equipment is either unavailable or limited by available resources, one may attempt alternative techniques such as enzyme-based treatment such as DNAse or dsDNA Fragmentase (e.g., NEBNext ds DNA Fragmentase), probe sonication, nebulization, or an optimized combination of these methods.
  a. Quantify extracted genomic DNA by using the Nanodrop Spectrophotometry (Thermo Fisher Scientific) or other spectrophotometric method. Nanodrop is quite suitable because it requires very small amounts of the biological samples.
  b. Fragment DNA in aliquots of 30 μg DNA in 130 μL of TE at the following conditions: Duty cycle – 10%, Intensity – 4, Cycles/burst: 200, time: 80 s.
  c. Analyze the fragmentation by electrophoresis (Figure 2A, TAE 2% low-melting- point agarose gel) and visualize the DNA by ethidium bromide staining. The gel should be poured out and run within a cold room or refrigerator to avoid melting.
  d. Observe and document the fragmented DNA under 320–365 nm UV light (UV A). Excise the DNA between the 100–500 bp range with a sharp surgical scalpel.
  e. Weigh the gel slice and add 2.5 volumes of TE buffer. Incubate at 65°C for 15 min or until the gel is completely dissolved. Vortexing every few minutes will help accelerate the process.
  f. Add an equal volume of 1:1 phenol:chloroform (vol/vol), vortex and then centrifuge it for 5 min at 8,000 × *g* before carefully discarding the upper organic phase.
  g. Repeat the previous step until no solids can be observed in the interface between the aqueous and organic phases.
  h. Add an equal volume of chloroform, vortex and centrifuge it for 5 min at 8,000 × *g*. Discard upper organic phase.
  i. Repeat the previous steps as needed until desired amount and purity are obtained (see below).
  j. Quantify purified DNA using Nanodrop. The yield of DNA recovery after precipitation varies from 50 up to 80%.
  k. Correct the DNA 5′ and 3′ ends by treatment with T4 DNA polymerase (Thermo Fisher Scientific); this is very important to generate the blunt-ends required for efficient cloning into the vector. Use 1 U of T4 DNA Polymerase per μg of DNA in the presence of 0.1 mM dNTP at 11°C for 20 min.
  l. Stop the reaction by heat inactivation (75°C for 10 min).
  m. Purify DNA using the QIAGEN PCR Purification Kit. Respect the recommended column capacity by adding only 10 μg of DNA to each column. To be able to purify small fragments (<300 bp), after mixing the sample with 5 volumes of the binding buffer (PB), add 2.5 volumes of 2-propanol and admix it thoroughly.
  n. Run 100 ng of the material in a 2% agarose gel. A smear within the desired region (100–500 bp) should be observed (Figure 2).

2. Library cloning
  At this stage, it is necessary to digest the phagemid vector and optimize ligation conditions by varying the vector:insert ratios. It is important to remember that 1 in 18 clones in your final library will (theoretically) display a peptide epitope that belongs to the proteome of the target organism (Figure 1C). So, this should be taken into consideration in order to properly estimate the library diversity (unique sequences) and genome coverage (average number of genomic-fold coverage).
  Day 1:
  a. Digest the chosen phagemid vector with the restriction enzyme. If using the pG8SAET vector, use *Eco105I* (Thermo Fisher Scientific) following the standard manufacturer protocol.
  b. Purify the digested vector by phenol:chloroform extraction and ethanol precipitation or an alternative methodology, such as silica column purification (e.g., Qiagen PCR purification kit).
  c. Perform ligations (Table 1) with T4 DNA ligase (Thermo Fisher Scientific) and different vector:insert ratios. To optimize vector:insert ratio, test at least three different ligation conditions using between 50 and 300 ng of insert for 100 ng of the restriction enzyme digested vector. Incubate the ligation reaction at 16°C for 16–24 h. These ligation conditions will roughly correspond to vector:insert molar ratios of 1:5, 1:10, 1:20, and 1:30 (mol/mol) (Figure 2B).

**Table 1:** Ligation reaction

Reagent	Quantity
pG8SAET phagemid digested	100 ng
Blunt-end genomic fragments	50, 100, 200 or 300 ng
10**×** T4 DNA ligase buffer	To 1**×**
PEG 50%	To 5%
T4 DNA Ligase	5 U
    Day 2:
  d. Precipitate the ligations by adding 3 volumes of ice-cold ethanol, 0.1 volumes of 3M sodium acetate pH 5.2 and centrifuging at 20,000 × *g*, 4°C for 30 min. Wash with 70% ethanol, then centrifuge for 10–15 min to pellet the DNA (this is important to remove excess salt). Resuspend each ligation in 10 μL of ddH_2_O.
  e. Transform each ligated DNA in 100 μL of *E. coli* DH10B strain by electroporation using a 2-mm gap cuvette (2.5 kV, 200 Ω, 25 μF), and recover in 1 mL of outgrowth media. Culture them at 37°C for 1 h, with agitation (250 rpm). Plate 10-fold serial dilutions (1/10, 1/100 and 1/1,000) in LB agar plates (duplicates) containing 50 μg/mL of carbenicillin plates to estimate the number of total transformants obtained.
    Day 3:
  f. Calculate the total number of transformants for each condition based on the number of host bacterial colonies obtained and the dilution factor.
  g. With the optimal conditions and molar ratios empirically determined, scale up the ligation and transformation procedure accordingly to obtain the desired number of transformants (see below and item 5: gPhage display library quality control). In brief, the number of transformants multiplied by the average length of the inserts has to yield a number that is at least 100-times the length of the genome of the organism (ideally, >1,000-times).
  h. After transformation and culture outgrowth for 1 h, pool all transformed bacteria into one tube. Transfer 100 μL to a tube containing 900 μL of LB and perform 10-fold serial dilutions. Plate 100 μL of each dilution in carbenicillin containing agar plates (50 μg/mL) (duplicate). Incubate the plates at 37°C for 16–20 h. This procedure allows for calculating the total number of transformants.
    ***Note:*** It is essential to perform colony counting at this time. After 12–16h culture (item i), your culture will be saturated and it is no longer possible to calculate the number of transformants.
  i. Transfer the bacteria to 500 mL of liquid media (LB 50 μg/ul carbenicillin), and grow at 37°C for 12–16 h, at 250 rpm.
  j. Next day, purify plasmid DNA by QIAGEN Maxiprep kit and CsCl (or your preferred alternative methodology), re-suspend it in TE buffer (Tris 10 mM, EDTA 1 mM, pH 8.0) and keep it stored at −20°C. The resulting reagent is a *T.cruzi* gPhage phage library, that can be used to produce peptide-displaying phage particles for selections. It is expected to obtain 200–500 μg of the library DNA.
  k. Calculate the total number of transformants in our library based on the number of host bacterial colonies obtained and the dilution factor. To assess the quality of your library, follow the steps below.

***Note:*** Although we have not attempted, using a vector that has the ccdb1 gene as a stuffer may significantly reduce background colonies (with empty vector) and be a time saver as one does not need to purify the backbone after digestion.

3. Library quality-assurance/quality-control
  a. Assess library quality control by colony Polymerase Chain Reaction (PCR): pick random 96 (or more) bacterial colonies infected with the gPhage library and perform colony PCR following polymerase manufacturer instructions (e.g., for ThermoFisher recombinant Taq polymerase, 95°C**×**3 min; 35 cycles: 95°C**×**20 s, 55°C**×**30 s, 72°C**×**1min30 s; 72°C**×**5 min, 12°C **×** hold) and Sanger DNA sequencing primers (Table 2). Use the empty vector pG8SAET as control (undigested and single-cut vector). Run samples in an agarose gel and calculate insert size (Figure 2). Comparison with the pG8SAET vector control should reveal whether the clones contain inserts.

**Table 2:** Primers used for pG8SAET colony sequencing and PCR

Primer	Sequence
pG8SAET Sequencing Forward Primer	CAGGGGGTATTAATTTGAAAAGG
pG8SAET Sequencing Reverse Primer	TATTCGGTCGCTGAGGCTTG
  b. Quality assurance and quality control (QA/QC) the resulting gPhage display by DNA sequencing (Table 2): Pick 30–100 (or more) random bacterial colonies transformed with the gPhage library and sequence the insert by the Sanger sequencing method. Calculate insert length, verify the presence of open-reading frames (ORF) in each insert and make sure the DNA aligns to the genome of the species of interest.
  c. Assess library quality by Next-Generation Sequencing (NGS): follow protocols below for Illumina Miseq DNA sequencing and assess insert size distribution, the presence and number of ORF(s), and annotated assignment to reference genomes and/or proteomes.

***Note:*** if resources are limited, it is possible to perform the QA/QC of the gPhage phage library by NGS together with the biopanning selection samples by using DNA bar-coding. If so, it is important to perform the steps described in items a and b to ensure the library yields at least 100-fold coverage of the genome of the organism. It is also recommended that at least 10–20 colonies are sequenced by Sanger (item b) for QA/QC your library.

4. Library phage amplification and titration
  Day 1: Transformation and superinfection
  a. Transform 10 μg of the gPhage library DNA by electroporation into *E. coli* TG1 strain (Lucigen, also commercialized by Merck). Transfer the transformed bacteria to a new tube. Plate 10-fold serial dilutions to assess the number of transformants as previously explained for DH10B strain (section 2.h) and to ensure it is larger than the original library diversity. For example, if the gPhage library contains 10^8^ unique inserts, you should expect at least 10^10^ transformants. In contrast to DH10B cloning strain, TG1 has a F′ genotype that enables superinfection by the helper phage and is also an amber codon suppressor strain that allows pVIII protein mounting when using pG8SAET system (there is amber codon between the genomic DNA insert and the pVIII gene).
  b. Recover your pool of bacteria in 500 mL of LB media and grow for 1 h at 37°C, at 250 rpm. After one hour, add carbenicillin to 50 μg/mL. Grow your pool of transformed bacteria at 37°C (250 rpm agitation) until *log*-phase (OD_600nm_–0.4–0.8).
  c. Add 5**×**10^12^ plaque-forming units (pfu) of M13KO7 helper phage (New England Biolabs) at a final Multiplicity of Infection (MOI) of 20, and incubate it by shaking at 37°C for 1 h. To calculate the MOI, consider that an OD_600nm_ of 1.0 represents approximately 10^9^ cells/mL. For 500 mL, a total 2.5 cells × 10^11^ cells are expected and 5**×**10^12^ pfu of M13KO7 helper phage corresponds to and MOI of 20.
  d. Add kanamycin to a final concentration of 25 μg/mL and grow for 16–20 h at 37°C, 250 rpm.
    Day 2: Phage precipitation
  e. Phage particles are purified from the cultured media using the PEG/NaCl method (Giordano et al., 2001). In brief, remove bacterial cells by centrifuging the bacterial culture at 7,000 × *g* for 15 min; recover supernatant and add 0.15 vol of PEG/NaCl (see materials and equipment section). Incubate on ice for 1 h.
  f. Centrifuge at 8,000 × *g* 4°C for 30 min to pellet the phage particles and discard the supernatant.
  g. Re-suspend the resulting phage pellet in 10 mL of PBS. It helps to let it shake for 15 min at 37°C (250 rpm) and then clarify the phage solution by centrifuging the phage admixture at 7,000 × *g* for 15 min to remove residual bacterial debris.
  h. Repeat the procedure. Recover the supernatant and add 0.15 vol of PEG/NaCl, incubate for 1 h at 4°C or on ice and centrifuge at 8,000 × *g* at 4°C for 20 min.
  i. Discard the supernatant and re-suspend the phage pellet in 1 mL of PBS 50% glycerol (one may need to increase the volume if the resulting suspension is too viscous). Again, it helps to solubilize the phage particles by incubating for 15 min at 37^o^C (water bath).
  j. Centrifuge the phage admixture at 20,000 × *g* for 5 min to remove bacterial aggregates and debris.
  k. Recover the supernatant, aliquot the gPhage library and store it at −20°C until use.
    Day 3: Library titration
  l. Thaw an aliquot of your gPhage library and make serial dilutions (from 1/10^7^ to 1/10^9^).
  m. Grow one colony of *E. coli* TG1 strain in 10 mL of TB phosphate medium until OD_600nm_ reaches ∼ 0.4–0.8.
  n. Mix 200 μL of the bacteria with 20 μL of the diluted phage aliquots and incubate at 23°C–25°C for 30 min.
  o. Plate infected bacteria in triplicate in LB agar (50 μg/mL carbenicillin). Incubate at 37°C for 16–20 h.
  p. Next day: Count the host bacterial colonies and calculate the resulting titers in transducing units (TU) with the appropriate dilution factors.

5. Library QA/QC

A suitable gPhage display library has to cover at least 100-times the genome of the organism. This is because a substantial number of inserts will produce phage particles that do not display a peptide or will display peptides that correspond to an alternative reading of the genome (Figure 1). Ideally, the gPhage display library should have a coverage greater than 1,000-fold the genome (Teixeira et al., 2021). Initial estimates may be obtained by calculating insert sizes based on PCR products and then by sequencing them (Sanger, NGS or both). Select random clones from the plates used to calculate the number of transformants, perform PCR reactions and analyze the samples side-by-side with the control empty vector to estimate insert size. Ultimately, a far more precise estimation can be achieved by using NGS sequencing. However, if resources are limited, the quality of the gPhage display library may be initially assessed by PCR and agarose gel estimates followed by Sanger DNA sequencing of 30–100 or so positive clone sample size to confirm that DNA inserts belong to the infectious organism of interest. Then, once the biopanning selection is complete, the gPhage display library may be further analyzed by NGS along with the remaining samples. Once you have information on the size distribution of the clones, one may perform the following mathematical calculations to estimate the coverage of your library in reference to your target genome:

Librarysize=Averageinsertsize(bp)∗NumberofTransformants

$$Library\;coverage=\frac{Library\;size}{Genome\;size\;\left(bp\right)}$$

Illustrating here for the *T. cruzi* Sylvio X10 library (4.4 **×**10^7^ bp genome):

$$Library\;size=143\;bp*\;4.4\;x10^{8}\;transformants=6.29\;x10^{10}\;bp$$

$$gPhage\;display\;library\;coverage=\frac{6.29\;x10^{8}\;bp}{4.4x10^{7\;}bp}=1,430x\;coverage$$

For the vector and cloning method reported here, only 1 in 18 inserts should, in theory, yield a phage particle that in fact display a *T. cruzi* peptide (Teixeira et al., 2021). If so, the theoretical library coverage would be: 80-times the encoded proteome.

### gPhage display library selection

**Timing: 2 weeks**

After gPhage display library construction, donor selection and IgG purification from serum samples, one is ready to start the selections. For that, gPhage library is exposed to the immobilized IgG molecules to allow for phage particles displaying specific antigens recognized by the IgG to be captured. After washing away the unbound phage, the remaining phage particles bound to the IgG are recovered by infection with bacteria and amplified for further rounds of selection. It is important to note that we recommend adding excess control IgG during the selection step to prevent binding of phage to IgG constant regions or the selection of ubiquitous antigens. See Teixeira et al., 2021 (Fig. 2A within the reference) for an illustrative image of the epitope selection procedure for a *T. cruzi* library in the setting of patients with Chagas disease.

6. IgG immobilization and gPhage library selection
  Day 1: IgG coating
  a. Immobilize purified IgG (1 μg in 100 μL of PBS) on 96 wells microtiter high-binding enzyme-linked immunosorbent assay (ELISA) plate. Incubate the plate at 4°C for 16–24 h. Start by preparing a culture of *E. coli* TG1 bacteria by adding one colony in 10 mL of TB phosphate medium. The bacterial culture should be in *log*-phase (OD_600nm_–0.4–0.8) for the elution of phage bound to IgG - item e below).
    ***Note:*** It is also possible to perform the selection on samples of individual patient; in that case, we recommend the use of protein-G or anti-IgG (Fc fragment) pre-coated microtiter plates to directly capture the patient-derived IgG from sera samples. This step will markedly reduce the required volume of biological sample(s) used in the experiment.
    Day 2: First round of selection
  b. Wash wells 4-four times with PBS 0.1% Tween-20 to remove excess IgG. Block wells with PBS containing 1% bovine serum albumin (PBS/BSA) for 1 h at 23°C–25°C.
  c. Add the gPhage display library in 100 μL of PBS/BSA plus 10 μg/mL of IgG from your control group. The presence of the control IgG in solution is essential to prevent selection of ubiquitous antigens. Incubate for 2 h at 23°C–25°C. Regarding the amount of gPhage library added, see note below.
  d. Wash the wells ten times with 200μL of PBS.
  e. Add 100 μL of your TG1 bacteria (*log*-phase) in each well and incubate for 30 min at 23°C–25°C.
  f. Recover infected bacteria and dilute in 10 mL of LB broth containing carbenicillin (50 μg/mL).
  g. Take two or three small aliquots (10 μL each) from the 10 mL suspension and perform 10-fold LB broth serial dilutions. Plate the dilutions in LB agar 50 μg/mL carbenicillin plates and incubate at 37°C. This will allow to calculate how many phage particles were recovered in each round of selection to monitor enrichment (see Note below).
  h. Grow for 2 h at 37°C at 250 rpm.
  i. Superinfect cells with the helper phage by adding 10^10^ pfu of M13KO7. Incubate cell culture at 37°C for 1 h, with agitation (250 rpm).
  j. Add kanamycin to a final concentration of 25 μg/mL. Grow it at 37°C for 16–20 h, with agitation (250 rpm).
  k. To prepare for the next round, repeat the IgG immobilization step performed at the beginning of the procedure (item a).
    ***Note:*** The quantification step may be skipped entirely during the first round of selection in order to avoid loss of phage clones: phage clones that are removed from the 16–20h culture and plated for quantification, are not amplified in the LB broth, and, therefore, will not be present in the successive rounds of selection. When using a gPhage library with a high coverage (>1,000-times the genome), we find that this step is not a cause of concern due to insert redundancy (Teixeira et al., 2021).
    Day 3: Precipitation of first-round phages
  l. Centrifuge the 16–20 h cell culture for 10 min at 10,000 × *g*. Transfer supernatant to a new tube and add 0.15 volumes of the PEG/NaCl solution to precipitate phage particles. Incubate on ice for 30 min.
  m. Extract plasmid DNA from the remaining cell pellet. We recommend QIAprep Spin Miniprep Kit (or another method of your preference).
  n. Count the bacterial colonies of your recovered gPhage plated on the previous day and calculate the number of phage bound to each IgG sample by correcting with the dilution factor and the volume of your culture (10 mL).
  o. Centrifuge gPhage suspension 10 min at 10,000 × *g*. Discard the supernatant and resuspend the phage pallet in 1 mL of PBS/BSA.
  p. Centrifuge for 5 min at 20,000 × *g* to remove remaining bacterial cells and debris. Transfer supernatant to a clean microtube to be used for a new round of selection.
  q. Repeat selection procedure for a total of three rounds. Titrate both, the input and output phage for each round. An increase in the ratio output/input is a good indication of positive selection. If necessary, perform a fourth round. However, we find that further rounds of selection often do not improve results due to the takeover of fast amplifying phage particles and loss of diversity.

***Note:*** The amount of phage input for the first round should be at least 10-times the diversity of the gPhage library. This will assure a fair representation of most phage inserts present in one’s library. However, for the remaining rounds of selection, phage input can be adjusted. In our case (Teixeira et al., 2021), we performed two independent biopannings. Although we used similar amounts of gPhage library during the first round (∼ 10^9^ TU) for both selections, we varied the amount of phage during the second and third rounds of selection. This led to a significant difference in the overall number of antigens/epitopes identified. For details, see the discussion of the article.

### Sequencing and bioinformatic analysis

**Timing: 2 weeks**

7. Next-Generation DNA Sequencing
  In this step, plasmid DNA obtained from the naïve (unamplified, unselected) library or purified from each successive rounds of selection is used to generate amplicons suitable for Illumina MiSeq sequencing. The addition of variable length degenerations (Table 3) between the region of interest and the partial Illumina adapters removes the need for PhiX spike-ins to add diversity to the sequencing pool – this original step increases the number of reads that may be recovered after a sequencing run by 10–25% depending on the protocol used. Specific primers for pG8SAET containing degeneration and Illumina adapters are listed in Table 3 and are used for the first PCR step (which adds partial Illumina adapter sequence). It is also important to use a high-fidelity polymerase such as NEB Q5 or Roche Kapa HiFi. A similar protocol is also detailed in a previous work of our group (Tang et al., 2019).
  a. Use 10 ng of the plasmid DNA from the gPhage display selection and the unselected library as the template for your first PCR reaction and follow the DNA polymerase manufacturer instructions for the PCR (for KAPA HiFi, 95°C × 3 min; 20 cycles: 98°C**×**15 s, 58°C**×**15 s, 72°C**×**45 s; 72°C**×**2 min; 12°C **×** hold). Mix all 8 primers (4 forward and 4 reverse) in each reaction.
  b. Purify the PCR products by using silica columns (e.g., QIAquick PCR Purification Kit).
  c. Perform the second PCR reaction with the Nextera XT kit (Illumina) primers to add the indexation (for KAPA HiFi, 95°C **×** 3 min; 8 cycles: 98°C**×**15 s, 55°C**×**15 s, 72°C**×**45 s; 72°C**×**2 min; 12°C **×** hold). Follow the standard manufacturer protocol to prepare and quantify your sample. Quantification by qPCR at this step will help adjust library concentration (Library Quantification Kit, Kapa Biosystems). We recommend a 4 nM final concentration of your sequencing library.
  d. Sequence your samples using the MiSeq Reagent Kit v2 (500 cycles) on an Illumina MiSeq equipment. Other platforms, such as NovaSeq (SP 2**×**250 cycles Illumina), can be used for increased throughput. Depending on the expected insert length, one can choose to use a different number of cycles (e.g., 2**×**150 bp), but at the cost of losing longer sequences.

**Table 3:** Recommended sequence of primers for first PCR (pre-indexation)

Primer	Sequence		
	Partial Illumina index	Degenerated nt	pG8SAET annealing region
Fw-0N	5′-TCGTCGGCAGCGTCAGATGTGTATAAGAGACAG-		-CTGCGCAACACGATGACC-3′
Fw-1N	5′-TCGTCGGCAGCGTCAGATGTGTATAAGAGACAG-	-N-	-CTGCGCAACACGATGACC-3′
Fw-2N	5′-TCGTCGGCAGCGTCAGATGTGTATAAGAGACAG-	-NN-	-CTGCGCAACACGATGACC-3′
Fw-3N	5′-TCGTCGGCAGCGTCAGATGTGTATAAGAGACAG-	-NNN-	-CTGCGCAACACGATGACC-3′
Rv-0N	5′-GTCTCGTGGGCTCGGAGATGTGTATAAGAGACAG-		-CTGCGCAACACGATGACC-3′
Rv-1N	5′-GTCTCGTGGGCTCGGAGATGTGTATAAGAGACAG-	-N-	-CTGCGCAACACGATGACC-3′
Rv-2N	5′-GTCTCGTGGGCTCGGAGATGTGTATAAGAGACAG-	-NN-	-CTGCGCAACACGATGACC-3′
Rv-3N	5′-GTCTCGTGGGCTCGGAGATGTGTATAAGAGACAG-	-NNN-	-CTGCGCAACACGATGACC-3′

***Note:*** The PCR reaction should be limited to the minimal number of cycles possible to avoid introduction of point mutations and/or amplification-induced biases in our samples. We used 20 cycles for the first PCR and 8 for the second. We recommend the KAPA Hifi Hotstart ReadyMix 2**×** (Roche) or equivalent for PCR.

**Figure 3:**
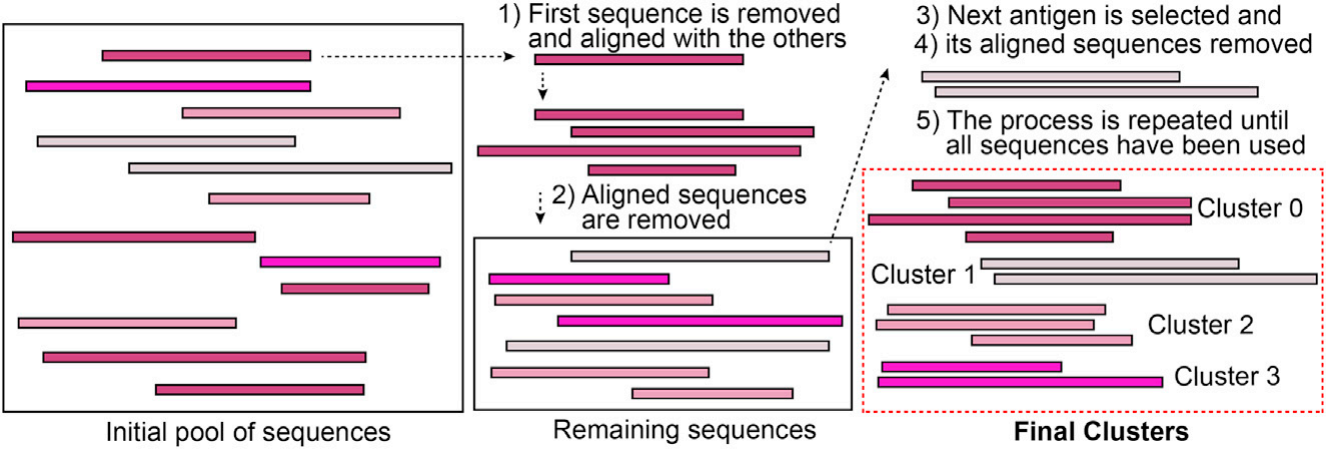
Clustering procedure Representation of the clustering algorithm to be used for antigen identification from third-round phage sequences (obtained by NGS) selected by the patient immunoglobulins (IgG).

8. NGS Data Analysis
  Sequencing data analysis is done in two steps. In the first step we process raw reads, from which the peptide sequences are inferred. In the second step we process the peptide sequences to generate a nonredundant peptide sequence dataset. The reason for the second step is the following: If a gPhage display library has high coverage, several slightly different peptides that correspond to the same epitope may be recovered. In general, it means that peptide sequence clustering is necessary to generate a nonredundant epitope/antigen sequence dataset. All files necessary to run the pipeline have been supplied (Data S1 and S2) or can be downloaded from GitHub (https://github.com/aarteixeira/gPhage/) (preferred, in order to use the most up-to-date version of the scripts). To run the pipeline, follow these steps (Figure 3):
  a. Assemble the paired-ends with PEAR; tools such as FastP (Chen et al., 2018) and PandaSeq (Masella et al., 2012) may also be used for this step as well.
  b. The assembled FastQ files can then be processed by using the script dna_processing.py (Data S1) to extract insert sequences and align them to the reference genome and/or proteome, as follows.
    i. Make sure you have Python3 software installed with the packages Pandas (v1.3.0), Biopython (v1.79) and Numpy (v1.21.0).
    ii. Make sure you have BlastN and BlastP (Camacho et al., 2009) installed for local pairwise alignment, and change the variable *blastn_exe* and *blastp_exe* in the script to reflect the path to the executables.
    iii. Using Blast software, create databases for your reference genome and proteome and change the *database* variable to reflect the path to the file.
    iv. Change the *dict_sample_fastq* variable (Table 4) to the name of your samples and the path to the respective FastQ file. Add as many samples as needed.

**Table 4:** Variables that can be customized at the script dna_processing.py

Variable	Description
BLASTN_EXE	path/command to BlastN executable
BLASTN_DB	path to BlastN database
BLASTP_EXE	path/command to BlastP executable
BLASTP_DB	path to BlastP database
dict_sample_fastq	sample names and path to respective FastQ to be processed. To add a sample, add a new line with the following code: dict_sample_fastq["Sample Name"] = "path_to_fastq.fastq"
cutoff_dna_identity	minimum relative identity to reference genome for a DNA insert to be considered a hit (default is 0.9)
cutoff_peptide_similarity	minimum relative similarity to reference proteome for a peptide to be considered a hit (default is 0.6)
flank_upstream	sequence found upstream of the DNA insert (default set for pG8SAET sequence)
flank_downstream	sequence found downstream of the DNA insert (default set for pG8SAET sequence)
    v. If you want to remove DNA sequences that appeared just once (‘singletons’), change the variable *option_remove_singletons* to *True.* This setting is recommended only for your samples and not for the gPhage naïve library, since most sequences will be unique.
    vi. To remove DNA sequences that do not match the reference genome, change *option_remove_DNA_below_the arbitrary cutoff* to *True* and set the minimum identity at *cutoff_dna_identity* (we currently recommend 90% or higher).
    vii. To remove peptide sequences that do not match the reference proteome, change *option_remove_peptide_below_cutoff* to *True* and set the minimum identity at cutoff_peptide_similarity (we currently use 60% or higher).
    viii. Run the script: “python3 dna_processing.py” to obtain de output files (Table 5).
      ***Note:*** Another consideration is whether the bacteria you have used to amplify the library is a suppressor strain. For TG1 bacteria (amber suppressor), the codon TAG codes for a glutamine instead of the usual stop codon. Our script takes this change into consideration.

**Table 5:** Output files generated by the script dna_processing.py

Output file	Description
dna_dataframe.xlsx	table containing all DNA inserts identified in all samples, their respective frequencies in each sample and BlastN results against reference genomes.
dna_above_cutoff.xlsx	same as dna_dataframe.xlsx, but containing only the inserts that match the reference genomes above the BlastN identity cutoff
dna_below_cutoff.xlsx	same as dna_dataframe.xlsx, but containing only the inserts that are below the BlastN identity cutoff (or do not match reference genomes)
peptide_dataframe.xlsx	table containing all peptides identified in all samples peptide, including the frequency in each sample and BlastP results against reference proteomes.
norm_peptide_df.xlsx	same as peptide_dataframe.xlsx, but with counts normalized by total DNA (relative frequency)
peptide_above_cutoff.xlsx	same as peptide_dataframe.xlsx, but containing only the peptides that match reference proteomes above the BlastP similarity cutoff
peptide_below_cutoff.xlsx	same as peptide_dataframe.xlsx, but containing only the peptides that are below the BlastP similarity cutoff (or do not match reference proteomes)
statistics.xlsx	general processing statistics
peptides.txt	A list of all peptides identified by the script. This file can used as input for the clustering.py script
  c. Having obtained all the information regarding the peptide sequences of interest, one may now generate clusters to characterize the epitopes using the script clustering.py (Data S2).
    i. Make sure you have Python3 installed with the packages Biopython and FuzzyWuzzy installed (Levenshtein, 1966).
    ii. Make sure you have the software MAFFT (Katoh and Standley, 2013) and HMMER (Mistry et al., 2013) installed and change the variables *MAFFT*, *HMMBUILD*, and *HMMEMIT* (Table 6) to reflect the path to the corresponding executables.

**Table 6:** Variables that can be customized at the script clustering.py

Variable	Description
PEPTIDES_TXT	path to the file containing the peptides to be clustered (text file containing one peptide sequence per line)
MAFFT	path/command to MAFFT executable
HMMBUILD	path/command to hmmbuild executable
HMMEMIT	path/command to hmmemit executable
K	K-mer length for peptide search. Only peptides that share a K-mer are compared. Default is 4 amino acids.
min_len	minimum length of the peptides used for clustering. Peptides with length below this threshold are excluded. Default is 6 amino acids.
score_cutoff	minimum score (% partial identity) for peptides to be clustered together. Default is 80.
    iii. Create a file named peptides.txt containing all peptides to be clustered (one peptide per line) in the same folder containing the script. If you used the dna_processing.py script, a “peptide.txt” was generated for you.
    iv. Change variables *K*, *min_len*, and *score_cutoff* to alter the K-mer length for comparison (default is 4), minimum length of a peptide to be considered (default is 6) and minimum percentage similarity between two peptides to be grouped (default is 80), respectively.
    v. Run the script: “python3 clustering.py” to obtain the output files (Table 7).

**Table 7:** Output files generated by the script clustering.py

Output file	Description
cluster_consensus.xlsx	table containing cluster IDs, number of different peptides forming the clusters, and consensus sequence.
peptide_cluster.xlsx	table containing all peptide sequences and corresponding cluster.
align folder	folder containing the multiple sequence alignments, consensus sequences, and HMM profiles for each cluster.

9. Characterization of the gPhage library

You should determine the number of unique inserts and the average size of your inserts. It is necessary to determine the percentage of transformants that contain *T. cruzi* inserts. Considering the total number of transformants, one may use BLASTn to determine the percentage of clones corresponding to your target organism. The scripts used for library characterization are available in Data S1.

***Note:*** In our study, the gPhage library contained ∼4**×**10^8^ (as determined by the number of bacterial colonies when plating the library after initial transformation). In this step, the equation (see Library QA/QC step) is used to calculate the real coverage. NGS of ∼2.2**×**10^5^ sequences showed high diversity (99% unique sequences) with an average size of 143 bp and that 75% clones (∼3**×**10^8^) contained a *T. cruzi* insert. All this corresponds to a total of ∼4.3**×**10^10^ bp of the *T. cruzi* haploid genome (3**×**10^7^ bp) or ∼1,400 times. However, due to the blunt-end cloning strategy, inserts may be cloned in all possible reading frames and orientations. So, not all inserts result in peptide display and not all displayed peptides correspond to a *T. cruzi* protein (Figure 1**C**, some peptides may be derived from alternative reading frames of the genome). Taking this into consideration, only 17.8% of the inserts in our gPhage library encode a peptide and among those, only half (8.9%) share some degree of similarity (60%) with a *T. cruzi* protein.

### Identified epitope validation

**Timing: 3 weeks**

To validate identified epitopes one may choose two alternative routes: (i) To use synthetic peptides and direct ELISA assays or, (ii) To perform sandwich ELISA or FLISA (Fluorescence-linked immunosorbent assay) assays by using gPhage particles displaying the epitope. The first option is straightforward and may allow the validation of linear epitopes, granted that the synthetic peptide binds the ELISA plate. However, for most epitopes (including but not limited to conformational ones) and those in which the synthetic peptide does not adhere to plastic, one can use an indirect approach in which the donor IgG is first coated onto the ELISA plate, incubated with the gPhage particles displaying the peptide of interest, and finally detected by using a secondary reagent (Sandwich gPhage-based assays). This option has the advantage that the peptide will retain its original conformation as displayed on the phage particle.

10. Direct ELISA validation with peptides

The NGS data generated is a useful starting point to select candidates. Epitopes highly enriched against the patient serum samples of interest but not against the control serum samples are more likely to be true hits. Often times, the most abundant antigens also contain the highest number of overlapping peptides, which may be used for epitope mapping by identifying the conserved core sequence. A synthetic peptide may then be designed and custom-ordered for validation assays.

**Figure 4:**
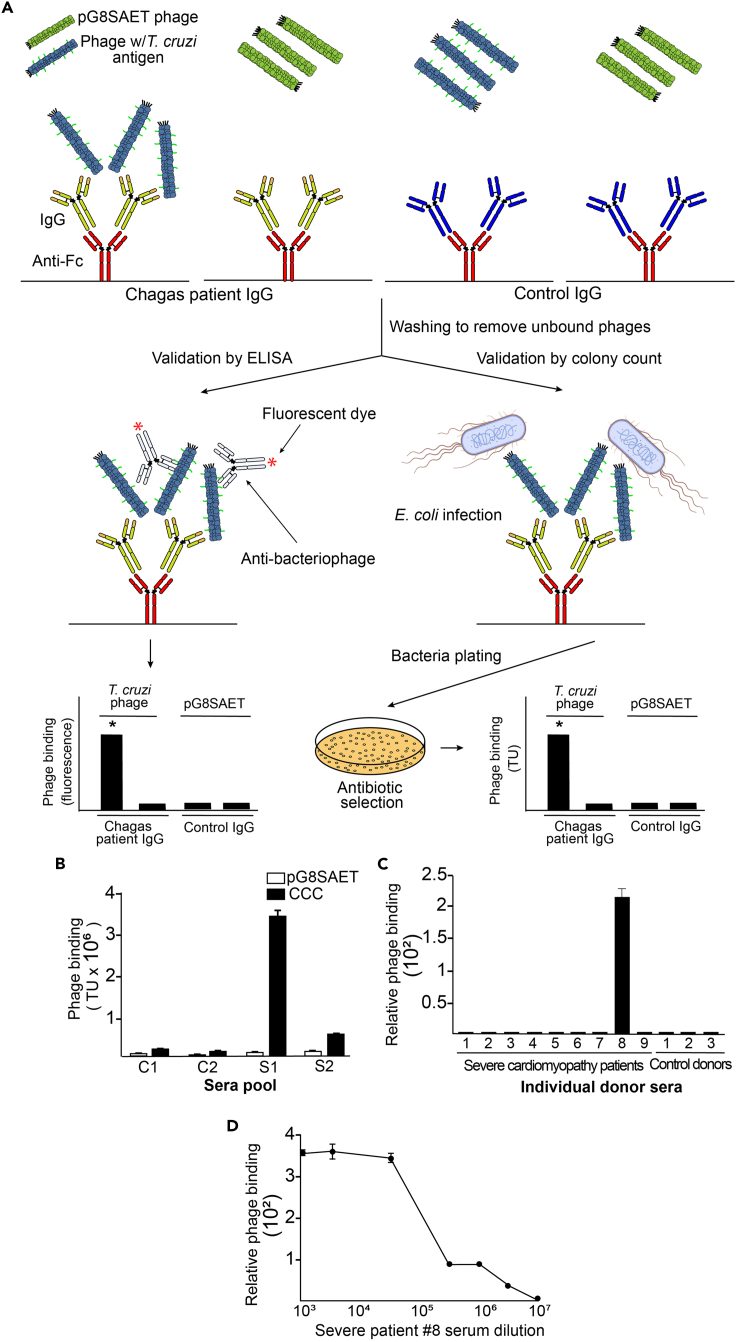
A gPhage-based validation assay (A) Scheme to illustrate the phage binding assay applicable to validate the specific binding of epitope-containing phage to patient immunoglobulins. The number of colonies obtained after plating and incubation can be multiplied by the dilution factor to obtain the Transducing Units (TU, viable phage particles) value. The validation can also be performed by immunoassays with an anti-bacteriophage antibody labeled with HRP or a fluorophore, for example. (B) Phage binding assay for a specific phage obtained when performing the panning with a pool of severe cardiomyopathy Chagas disease patients. For this binding assay, two pools of sera from patients with severe cardiomyopathy (S1 IgG, S2 IgG) and two pools of control sera (C1 IgG, C2 IgG) were used. Phage particles (CCC phage) show strong binding to immunoglobulins of patients with severe cardiomyopathy in comparison to the corresponding binding to negative control insertless phage particles (pG8SAET). (C) Relative phage binding value (quotient of the CCC and pG8 binding values in Transducing Units for each point) when performing the binding assay with individual patient serum samples. An index (S8) patient serum sample presents a 200-fold CCC/pG8 quotient, indicating a strong sensitivity when incubating the CCC phage particles with the IgG of this patient. The index patient serum was included in the S1 IgG and S2 IgG pools during the panning selection. (D) Reactivity of the CCC phage with index patient serial serum dilutions. High-reactivity is observed until 10^6^ dilution. In all cases, error bars represent SEM from biological (N=2) and technical (N=3) replicas.

Use standard ELISA to immobilize the peptide on the microtiter wells and test the reactivity against either purified IgGs or serum samples. However, in cases where reactivity is not detected, there is still the possibility that the synthetic peptide does not perfectly mimic the true epitope conformation and/or not adhering well to the plastic surface. Detailed results of the ELISA analysis for Chagas disease-related epitopes screening are shown in Teixeira et al. (2021) (Figure 4 in the article).

11. Sandwich gPhage-based assays
  This type of assays is useful to assess the reactivity of epitopes that require specific conformations for binding or peptides that may be hard to adhere to the microtiter plates. First, isolate a single bacterial colony with the gPhage displaying the antigen of interest. In cases where the antigen/peptide of interest has a high frequency in the pool of isolated phage particles, the corresponding monoclonal phage may be isolated by sequencing individual bacterial colonies by Sanger DNA sequencing. If that is not possible (i.e., low frequency phage clone), synthetic oligonucleotides encoding the antigen sequence may be ordered and subsequently cloned back into the phagemid vector pG8SAET. It may also be useful to produce phage particles from the empty phagemid – this step will yield particles that do not display any foreign peptides and will serve as a suitable negative control in the binding assays. This protocol can be performed by using purified IgG or by direct capturing the IgGs on the microtiter plate. Also, the quantification step may be performed either by using fluorescent/colorimetric secondary reagents or by rescuing the bound phage with host bacteria infection and plating serial dilutions for colony counting (Figure 4A).
  a. Monoclonal Phage Production
    i. In a 50 mL conical tube, inoculate a single bacterial colony of TG1 bacteria infected with the clone of interest into 10 mL of LB broth 50 μg/mL carbenicillin and grow at 37°C, with agitation (250 rpm) until early *log* phase (OD_600nm_ = ∼0.5).
    ii. Perform the helper phage superinfection, growth, precipitation, and titration of the phage produced as described at *“Library phage amplification and titration.”*
  b. IgG immobilization and gPhage binding
    ***Option A:*** Microtiter well coating procedure if working with purified IgG:
    i. Immobilize IgG (10 μg/mL in PBS) from individual donors onto 96-well High-Binding ELISA plates (100 μL/well), by incubation at 4°C for 16–24 h. Use triplicate wells for each clone to be tested, plus triplicate for each negative control.
    ii. Wash the wells four times with PBS (200 μL) and block with PBS/BSA for 1 h at 23°C–25°C.
    iii. Incubate with selected gPhage particle or the negative control (insertless phage, pG8SAET). Use 10^9^ TU of phage particles in 100 μL PBS/BSA for 2 h at 23°C–25°C.
    iv. Wash the wells four times with PBS
      OR
      ***Option B:*** Microtiter well coating procedure if working with donor serum:
    v. Immobilize goat anti-human IgG Fc (Jackson Immuno Research) (10 μg/mL in PBS) onto 96-wells of a High Binding ELISA plates by incubation at 4°C for 16–24 h (use 100 μL/well). Use triplicate wells for each clone to be tested, plus triplicate for each negative control (control from healthy donors and the goat-anti human IgG Fc with no added serum).
    vi. Wash the wells four times with PBS (200 μL) and block the wells with PBS/BSA for 2 h at 23°C–25°C.
    vii. Quantify using nanodrop (OD_280nm_) the protein concentration in your sera samples. Adjust the serum protein concentration to 120 μg/mL with PBS. Add 50 μL of diluted serum per well coated with anti-human IgG Fc. Incubate for 2 h at 23°C–25°C.
    viii. Wash the wells four times with PBS
    ix. Incubate with selected gPhage particle or the negative control (insertless phage, pG8SAET). Use 10^9^ TU of phage particles in 100 μL PBS/BSA for 2 h at 23°C–25°C.
    x. Wash the wells four times with PBS
  c. gPhage binding quantification
    ***Option A:*** Immunodetection (near-infrared fluorescence quantification):
    i. Incubate with the anti-fd bacteriophage antibody produced in rabbit (Sigma) for 1 h at 23°C–25°C (1:400 in PBS; 100 μL).
    ii. Wash the wells, incubate with IRDye 680LT Goat anti-Rabbit IgG Secondary Antibody for 1 h at 23°C–25°C (1:1,000 in PBS; 100 μL) (Licor Biosciences).
    iii. Wash the wells four times with PBS and proceed with the quantification step by using the Odyssey system (or equivalent near-infrared fluorescence quantification system).
      OR
      ***Option B:*** Quantification by colony counting:
    iv. Rescue phage form each well with 200 μL of *E. coli* TG1 in *log*-phase and infect at 37°C for 30 min.
    v. Make serial dilutions (from 1:10 to 1:1000) and plate in quadruplicate each well and incubate at 37°C for 16–20 h.
    vi. Next day: Count all bacterial colonies and plot the total TU of selected phage and compare with the control insertless phage (Figure 4).

***Note:*** Examples of these assays for the gPhage particle displaying the peptide motif PPHTRRVTVRCGPPSCADE vs. control phage particles (pG8SAET), selected by serial binding to the pool of serum samples from patients with Chronic Chagas disease Cardiomyopathy (CCC) patient pool (Teixeira et al., 2021) are shown in Figures 4B–4D.

## Expected outcomes

In general, constructing genomic libraries of complex organisms is technically cumbersome, labor- and cost-intensive procedures. The gPhage technology reported here allows for the constructions of relatively fast and cost-effective genomic phage display library of eukaryotes with no- or low-intron penetrance, viruses, bacteria or other organisms with or without available genomic information. This methodology is especially useful for the rapid identification of epitopes in emerging viral or neglected parasitic diseases. When applying this procedure, one should obtain gPhage libraries with high coverage to allow for unbiased selections. The libraries should include multiple copies of the same epitope, with difference in sizes that, in some cases, may facilitate the identification of large conformational epitopes. Although only a fraction of the initial gPhage display library phage particles encode true antigens from the organism of interest, upon selection against patient immunoglobulins, these out-of-frame phage particles work as an internal control: during selection, it is expected an enrichment of in-frame inserts that can then be clustered to define B-cell epitopes. The most abundant peptides can then be conveniently validated by ELISA and/or phage binding against the patient-derived immunoglobulins or without the need of expensive peptide synthesis by using the sandwich gPhage-based assay option. These even may result in some gPhage particles that may be used as diagnostic tools of the target disease.

## Quantification and statistical analysis

The standard software GraphPad Prism6 was used for graphical and statistical analysis. Student t-tests were used to compare results with control and testing phage particles (Figure 4A).

## Limitations

The amount of starting material (genomic DNA) from your target organism may limit your pipeline. Although the protocol may be followed in a straightforward manner, it is necessary to obtain a high coverage library to perform the selections. This could demand multiple vector ligation attempts. Besides, in the ligation step concatemers may form and artificially join two distinct genomic fragments into a single insert. Moreover, one is also limited by the size of the peptides being displayed: epitopes relying on long domains may not be identified when small fragments sizes are used. Even if using large fragments, there is no guarantee that the foreign sequences will be properly produced, folded, and displayed by the phage (because of many factors, including possible toxicity to the phage/bacteria). Finally, gPhage is better suited to organisms with low-intron content, but a similar approach may conceivably be used with cDNA instead, with the potential caveat that the library will be inherently biased by the genomic expression at the time of RNA extraction.

## Troubleshooting

### Problem 1

Insufficient fragmentation of genomic DNA.

### Potential solution

Increase duty cycle or intensity until you obtain a smooth smear of DNA with the desired range (100–500 bp) is visible by agarose gel electrophoresis (refer to step 1b).

### Problem 2

Not enough number of transformants after library ligation and electroporation.

### Potential solution

Test different vector:insert ratios (refer to steps 2c and 2 g).

It is always helpful to transform your non-digested empty vector (pG8SAET) to estimate the quality of your batch of electrocompetent bacteria. It should yield at least 10^9^ transformants per μg of plasmid DNA (refer to step 2e).

Increase the ligation time and use 5% PEG 6000 in your ligation mix (refer to step 2c).

Phosphorylate your fragmented insert with T4 polynucleotide kinase (refer to step 1 m).

If necessary, re-purify the vector and inserts again to remove any interfering contaminants (refer to steps 1 m and 2b).

### Problem 3

After optimization, my first library does not cover the whole genome (section 5 - Library QA/QC).

### Potential solution

Do not discard the library (refer to steps 3c and 5).

Repeat library construction with a larger amount of insert and vector (refer to step 2 g).

It is possible to combine two, three or more sub-libraries to produce a complete library in order to achieve at least 100-time genome coverage (ideally, >1,000-times) (refer to steps 3c and 5).

### Problem 4

Limited phage particle yields during each round of screening.

### Potential solution

Re-titer your helper phage and reassure you are applying the correct amount of plate forming units (refer to step 4c).

If necessary, prepare a new batch of helper phage (https://international.neb.com/protocols/0001/01/01/m13-amplification) (refer to steps 4c and 6i).

Check the antibiotic activity of the batches of carbenicillin and kanamycin in use (refer to steps 6f and 6j).

### Problem 5

Non-reactive synthetic peptide in ELISA for a highly enriched bacteriophage after selection.

### Potential solution

If possible, order a synthetic peptide containing biotin and use streptavidin conjugated to horseradish peroxidase to assess peptide binding to the microtiter plate. Alternatively, perform the sandwich gPhage-binding assay to validate the selected antigen (refer to step 10).

## Resource availability

### Lead contact

Further information and reasonable requests for resources should be directed to and fulfilled by the lead contact, Ricardo José Giordano (giordano@iq.usp.br).

### Materials availability

The vectors and reagents generated in the study are available from the lead contact upon reasonable request.

## Data Availability

Data used and codes generated can be found in Teixeira et al. (2021) and in this article supplemental information.

## Supplemental information

Data S1. Python scripts to process large-scale DNA sequencing data, related to step 2 (NGS Data Analysis)

Data S2. Python scripts for clustering the peptides, related to step 2 (NGS Data Analysis)
